# Characterization of the complete chloroplast genome of *Pyrus pyrifolia* ‘Yunhongli No. 1’

**DOI:** 10.1080/23802359.2020.1800429

**Published:** 2020-07-30

**Authors:** Longjie Cheng, Yuying Wang, Jin Yang, Yingyun He, Guochang Li, Weirong Ma, Xinglong Huang, Jun Su

**Affiliations:** a Institute of Horticulture, Yunnan Academy of Agricultural Sciences, Kunming, PR China; b College of Horticulture and Landscape, Yunnan Agricultural University, Kunming, PR China; c Industrial Crop Institute, Yunnan Academy of Agricultural Sciences, Kunming, PR China; d Station of Shi Lin Industrial Crop, Shilin, PR China; e Station of Hong He Industrial Crop, Mengzi, PR China

**Keywords:** Red pear ‘Yunhongli No. 1’, *Pyrus pyrifolia*, chloroplast genome, phylogenetic analysis

## Abstract

‘Yunhongli No. 1’ is a rare and well-colored red pear (*Pyrus pyrifolia*) germplasm resource, and is popular in the market due to its bright red color and high quality. This study used Illumina high-throughput sequencing technologies for red pear ‘Yunhongli No. 1’ of chloroplast genome sequencing and analysis. The genome features of *P. pyrifolia* ‘Yunhongli No. 1’ and the phylogenetic relationships were reported and established. The complete chloroplast genome is 160,113 bp in length, consisting of a pair of inverse duplication regions 26,386 bp, a large single-copy region 88,052 bp, and a small single-copy region 19,214 bp. The entire genome contains 80 messenger RNA (mRNA) genes, 30 transfer RNA (tRNA) genes, and 4 ribosomal RNA (rRNA) genes. The phylogenetic tree of 15 *Rosaceae* species revealed red pear ‘Yunhongli No. 1’ is more closely related to *Pyrus communis* × *P. pyrifolia* ‘Greensis.’

Red pear (*Pyrus pyrifolia*) ‘Yunhongli No. 1’ belongs to the pear subfamily (*Pomoideae*) of *Rosaceae*. ‘Yunhongli No. 1’ is a late-maturing variety, maturing in early October, and the 2/3 red coloration of the skin is an important feature of this fruit under natural conditions in Yunnan (Huang et al. 2010; Zhang et al. 2011). It originated from wild resources of Yanshan in the southeast of Yunnan Province, and was selected and bred by the Horticultural Research Institute, Yunnan Academy of Agricultural Sciences, and obtained a new variety certificate for Yunnan Province registration in 2003. In Yunnan Province, it is cultivated at elevations of 1700–2200 m where the temperature is 14 °C on average (Tao et al. 2003), annual precipitation is 890 mm, and annual sunshine is approximately 2000 h. Red skin of fruit is desirable in the market and plays a vital role in breeding.

The complete chloroplast genome sequences of red pear ‘Yunhongli No. 1’ was obtained (GenBank Accession No. MT598160). The genome sequences and features are significant to study the phylogenetic relationship of red pear ‘Yunhongli No. 1’ and helpful for the in-depth study of the chloroplast. Besides, it plays an essential role in the research diversity of genetic resources of this plant.

Test specimen of red pear ‘Yunhongli No. 1’ (*P. pyrifolia*) were gathered from the garden of the Yunnan Red Pear Science and Technology Development Company, Kunming, Yunnan (113.71°E, 34.71°N), and specimens were deposited in the Herbarium of Kunming Institute of Botany of CAS (specimen code: RP001). The trees had been grafted on the seedling of 7 years golden pear (*Pyrus betulaefolia Bunge* cv. Tang Li anvil) rootstocks. The improved CTAB method (Doyle and Doyle 1987) was used to extract the entire chloroplast DNA of red pear ‘Yunhongli No. 1’ from fresh mesophyll tissue.

Sequencing the DNA was performed using the Illumina NovaSeq in GENOSEQ Technologies Limited Company (Wuhan, China). Namely, the raw reads and clean reads were obtained and they were assembled by SPAdes (Dierckxsens et al. 2017). The assembled contigs were compared with the chloroplast genomes of the closely related species through the use of blastn version (BLAST 2.2.30+, parameter: -evalue 1e–5). Then the contigs were checked, selected, adjusted to get the final data. The chloroplast genome was annotated and mapped by using GeSeq (Tillich et al. 2017).

The length of complete chloroplast genome of red pear ‘Yunhongli No. 1’ is 160,113 bp. The genome presented a characteristic quadripartite circular structure which included one pair of inverted repeat regions (IRs, 26,386 bp), one large single-copy region (LSC, 88,052 bp), and one small single-copy region (SSC, 19,214 bp). Besides, the complete genome contains 80 messenger RNA (mRNA) genes, 30 transfer RNA (tRNA) genes, and 4 ribosomal RNA (rRNA) genes. The overall GC content of red pear ‘Yunhongli No. 1’ chloroplast genome is 36.56%. Moreover, the GC content of IR regions (42.65%) is higher than the LSC region (34.27%) and the SSC region (30.42%).

To study the phylogenetic relationship of red pear ‘Yunhongli No. 1’, a phylogenetic tree was constructed by using 13 complete chloroplast genomes of *Pyrus* species and two *Sorbus* species were selected as a outgroup. All the sequences were downloaded from NCBI GenBank. All sequences of species were aligned by the online program MAFFT version 7 and MEGA version 7.0 was used to build the maximum-likelihood phylogenetic tree with 1000 rapid bootstrap replicates (Kumar et al. 2016). The phylogenetic tree analysis indicated that *Pyrus communis* × *P. pyrifolia* ‘Greensis’ closely related to *P. pyrifolia* ‘Yunhongli No. 1’ (Figure 1).

**Figure 1.**
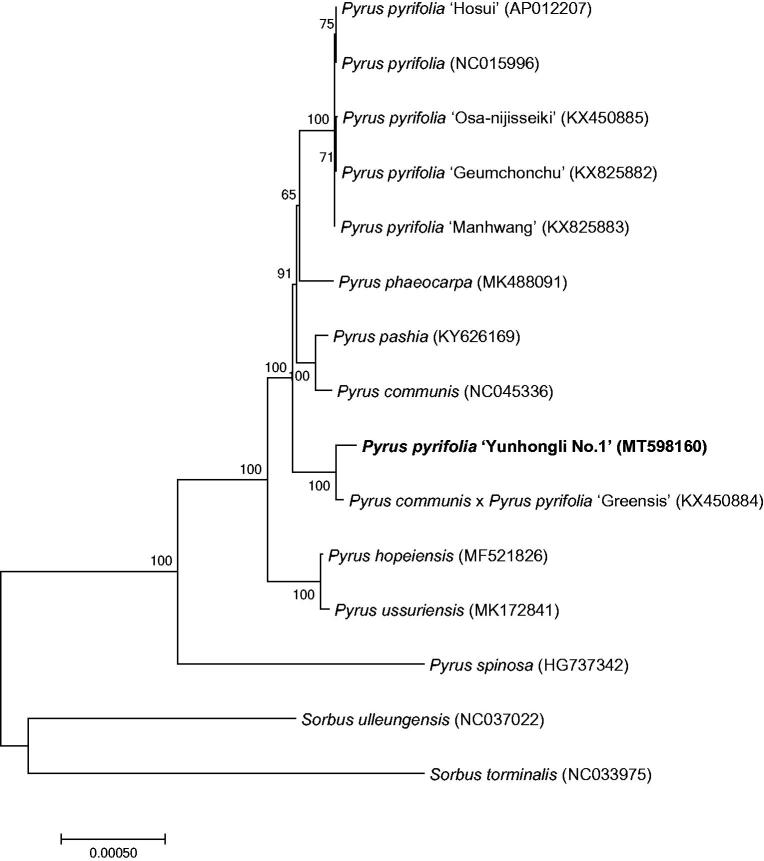
A Phylogenetic tree based on 15 complete chloroplast genome sequences of *Rosaceae* species using the maximum likelihood (ML) analysis by MEGA v7.0. Bootstrap support values are indicated in each node.

## Data Availability

The data that support the findings of this study are openly available in GenBank of NCBI at https://www.ncbi.nlm.nih.gov, reference number MT598160.
